# Exploring Video Consultations Across the Public and Private Sectors in Norway: Semistructured Interview Study

**DOI:** 10.2196/80812

**Published:** 2026-01-26

**Authors:** Mari Skoge, Sofie Ragnhild Aminoff, Henrik Myhre Ihler, Kari Jorunn Kværner, Linn Nathalie Støme, Kristin Lie Romm

**Affiliations:** 1 Early Intervention in Psychosis Advisory Unit for South East Norway Division of Mental Health and Addiction Oslo University Hospital Oslo Norway; 2 Institute of Clinical Medicine Faculty of Medicine University of Oslo Oslo Norway; 3 Department of Strategy and Entrepreneurship BI Norwegian Business School Oslo Norway

**Keywords:** clinician, hybrid psychotherapy, private sector, public sector, telemental health, telepsychiatry, therapist, video consultations, videoconferencing

## Abstract

**Background:**

Delivering therapy through video consultations can increase the reach and impact of mental health care services. However, adoption varies, and there is a lack of professional consensus about the usefulness of video consultations in therapy settings.

**Objective:**

This study aimed to explore mental health professionals’ experiences with and attitudes toward video consultations across different clinical environments in the private and public health care sectors in Norway to inform the design of future digitalized services.

**Methods:**

In this qualitative study, we recruited leaders and clinicians from public hospitals and private clinics. We conducted semistructured interviews that mapped individual experiences and attitudes concerning video consultations, as well as contextual aspects concerning the participants’ professional environments. We used reflexive thematic analysis with an inductive, essentialist, and experiential orientation to analyze the data.

**Results:**

A total of 24 mental health professionals (16 from public hospitals and 8 from private clinics) participated. Variations in their attitudes did not follow patterns reflecting the type of service or sector they worked in. Rather, attitudes seemed related to higher-level assumptions rooted in professional culture, societal values, and previous experiences. We generated six themes capturing and structuring the professional perspectives: (1) meta-perspectives on the digitalization of therapeutic rooms, (2) the “how” of service integration, (3) challenging therapist culture, (4) negotiating the limits of the digital therapy room, (5) creating clinical value from the digital format, and (6) adapting techniques and technology in digital therapy sessions.

**Conclusions:**

To strengthen the adoption and impact of video consultations, we should direct attention toward higher-level societal and cultural aspects that shape attitudes and practices. We suggest incorporating digitalized therapy in education, facilitating personal experiences with video consultations, increasing the sharing of knowledge between clinical environments, and sparking innovation of both service models and technology.

## Introduction

### Background

Implementation of video consultations (VC) as an alternative for mental health care delivery addresses several practical barriers to patients’ access to treatment [1-4]. The clinical outcomes of therapy delivered through VC are comparable to those of traditional in-person therapy for a wide range of mental health conditions, including depression, suicidal ideation, generalized anxiety disorder, and posttraumatic stress disorder [5-9]. Thus, studies investigating adaptations of psychotherapy practices to the VC format have emerged [10-14]. The literature on VC in services for severe mental illness and other less common mental health conditions is scarcer and inconclusive, but remains optimistic and encourages further research [15-19].

Hybrid therapy—the combination of VC and in-person therapy sessions—allows patients and clinicians to experience the benefits of both formats [20-24]. Previous research on the clinical use of VC has largely focused on comparing the service delivery alternatives in isolation. However, the flexible integration of both VC and in-person treatment modalities is increasingly treated as a promising approach for future mental health care services, potentially providing added value compared to using a single format [25-27]. Studies supporting hybrid models for delivering different types of clinical interventions have appeared in the literature [28-30], but there is a need for more research to develop ideal strategies for integrating VC as a component in routine hybrid services.

The adoption of VC varies significantly across mental health care [31,32], and the attitudes of mental health professionals contribute to this variation [33-36]. Some studies have shown that mental health professionals have largely positive attitudes toward VC, perceiving the format as acceptable and feasible for several clinical purposes [2,33,34,37-41]. Other studies have found greater diversity in perceptions of VC [42,43], and some have identified fundamental drawbacks reported by mental health professionals, such as feeling less present and experiencing a reduction in the quality of the therapeutic relationship [44-46]. Across studies, many report a lack of VC competency or an unmet need for support [14,35,36,47,48]. Furthermore, more experience with VC is associated with attitudes that are more positive [49-51]. Overall, mental health professionals show a preference for the traditional format [24,33,39], which is more evident than that of patients [37,48].

Organizational context is another factor that has been suggested as significant in influencing mental health professionals’ attitudes toward and intention to use VC [35]. The public and private health sectors represent distinct organizational conditions. In Norway, the number of private actors offering on-demand mental health care services has increased during the last decade, typically including digital treatment alternatives and opportunities for quicker access to services [52]. In parallel, the proportion of the population with private health insurance (as a supplement to the basic public coverage) has grown significantly [53], which influences the demand in the market for private health services. This development might indicate a more innovative climate, higher responsiveness to user demands, and quick adaptations to digitalization trends among private practitioners. However, we know little about how the private mental health sector approaches VC [54-56]. Health care systems are generally complex and characterized by organizational silos that challenge knowledge sharing across clinical environments [57-59]. Such gaps are highly noticeable within the public health care sector but are even more evident between the public and private sectors [60,61]. Another important reason for the lack of knowledge about private mental health actors is that the private sector in Norway typically has fewer incentives to spend resources on research activities, while the public sector finances and conducts most of the studies on mental health care services.

### Study Objectives

In summary, the topic of VC in mental health care lacks professional consensus. In this study, we aim to explore the experiences, attitudes, and contextual aspects related to mental health professionals’ use of VC. The study will include professionals working in various services across public and private mental health care to capture a broad range of perspectives. Our ambition is to develop knowledge about factors that contribute to differences in VC adoption and attitudes to inform the design of accessible future health care services that reach more people.

## Methods

### Study Design

This study is a qualitative investigation of mental health professionals’ experiences with and attitudes toward VC, as well as the professional contexts of the participants. The study uses a combination of convenience and snowball sampling strategies [62], digital semistructured interviews, and reflexive thematic analysis [63].

### Study Setting

The study was conducted in Norway. The Norwegian national public health system provides universal health coverage. The private health expenditure of the population is generally low (14% in 2021) [64]. Mental health care is provided through primary and specialized services, the latter requiring the presence of moderate or severe psychiatric conditions and a referral from the primary services. Specialized psychiatric services comprise both district psychiatric centers (local outpatient clinics) and hospitals. The services are semidecentralized and organized based on catchment areas, aiming to provide equal access to care across all social strata and geography [65].

The 2 public sector sites of our study are St Olav’s Hospital (Trondheim University Hospital) and Oslo University Hospital. The catchment area of St Olav’s Hospital consists of both urban and rural areas in Central Norway and is characterized by greater geographical distances between inhabitants and services than the catchment area of Oslo University Hospital, which serves urban areas only. The 4 private sector sites of the study are all located in major cities of Norway. These private clinics provide services to people who are self-referred. Their clients can pay for the services themselves or through private health insurance. Three of the private clinics in our study allow clients to book consultations directly through their websites, where clients can access information about available therapists and time slots. The fourth clinic requires clients to call, email, or submit a contact form. Two of the private clinics specialize in certain therapy orientations and patient populations, while the others offer more general mental health services. To protect the anonymity of the participants, we have chosen not to disclose the names of these clinics, as they are small, and the mental health professionals could be identified.

### Recruitment

The sample of participants in this study was obtained through convenience sampling, which included a snowball strategy [62]. We contacted the leaders of psychiatric clinics at St Olav’s Hospital, Oslo University Hospital, and several private clinics and invited them to take part as recruitment sites in our study (see Figures S1-S3 in Multimedia Appendix 1 for more details). The 2 public hospitals and 4 of the private clinics agreed to participate and approved the recruitment and data collection procedures. Two private clinics did not respond to our invitations. Both management and study participants at the clinical sites facilitated further recruitment of participants by informing colleagues about the project and sharing the project group’s contact information.

The inclusion criteria were as follows: (1) working as a clinician or working as a leader of other clinicians and (2) employed at one of the public hospitals or affiliated with one of the private clinics. All heads of the units had approved their participation and accepted that the participants spent an hour of their day on the interview. We did not aim to achieve a representative sample to generate representative findings; rather, we aimed to develop empirical thematic insights that were transferable to both research and clinical contexts [66]. We sought to access a broad range of viewpoints and contexts that would allow an exploration of whether expected characteristics (eg, sector affiliation) and other nonpredefined aspects influence approaches to VC. Importantly, we also included participants working in services targeting severe mental illnesses. A lesson learned from our previous work is that researchers and services should consider patients living with severe diagnoses as candidates for digitalized services, although they are often excluded from the target groups of such interventions [67].

### Data Collection

Data collection was carried out between May 2024 and December 2024. We conducted digital semistructured interviews addressing individual experiences and attitudes toward VC, as well as the participants’ workplace and professional environment. The interviews were based on an interview guide developed by the authors for the purpose of this study (Multimedia Appendix 2). We used the secure web-based VC platform Join, delivered by the state-owned company Norsk helsenett. The duration of the interviews ranged from 45 to 60 minutes. We recorded each interview with an app developed by Services for Sensitive Data (TSD), owned by the University of Oslo. The interviews were automatically transferred to the secure TSD platform and transcribed by the University of Oslo’s OpenAI Whisper technology.

### Data Analysis

We analyzed the interview transcripts from a critical realist position, applying a method largely based on Braun and Clarke’s [63] reflexive thematic analysis. We approached the data with an inductive, explorative, experiential, and contextualist approach [68]. The analysis was data-driven. It placed a direct focus on the participants’ utterances rather than on language and latent meaning. A contextualist orientation was a natural choice to capture the features of professional cultures and clinical environments. Choosing the reflexive school of thematic analysis, we aimed to transparently communicate the impact of researcher participation, as well as analytic decisions and processes. We selected this method with the ambition of developing meaningful themes that can carry transferable insights across the mental health field [66]. Moreover, the analysis was conducted from a pragmatic perspective that considered current and future clinical practices. We followed the 6 phases of thematic analysis outlined by Braun and Clarke [69], with some minor adaptations (Multimedia Appendix 3).

### User Representation

Two user representatives with lived experience, recruited through the Bipolar Association Norway, contributed to this study. The representatives provided feedback on the study in the early design phase and during the writing process. The purpose of this involvement was to ensure that the research aims, the methods applied to achieve these, and the angle used to present and discuss the findings are relevant, interesting, and valuable to people living with mental disorders, including severe conditions such as bipolar and psychotic disorders.

### Ethical Considerations

The project obtained approval from the data protection officer at Oslo University Hospital (24/04658). All participants provided written consent to take part in the study. The consent forms included information about the purpose of data collection and how the data would be stored and processed. The forms emphasized voluntary participation, including the option to leave the study at any time without risking any consequences. Signed consents and interview transcripts were stored in the secure TSD database. To protect the privacy of participants, identifiable features of participants were removed by MS within the secure TSD environment before the data were exported for analysis.

The economic compensation offered only to the participants working in private clinics entails ethical consideration. Importantly, private practitioners’ financial models conflict with involvement in research, which contributes to the maintenance of a knowledge gap concerning private health care services. Moreover, the project group holds the view that the compensation was not high enough to create any form of pressure for the private practitioners to participate in the study, only enough to compensate for lost income during the interview. Therefore, we evaluate the economic compensation to the participants working in private clinics as both ethically acceptable and reasonable.

## Results

### Overview

**Figure 1:**
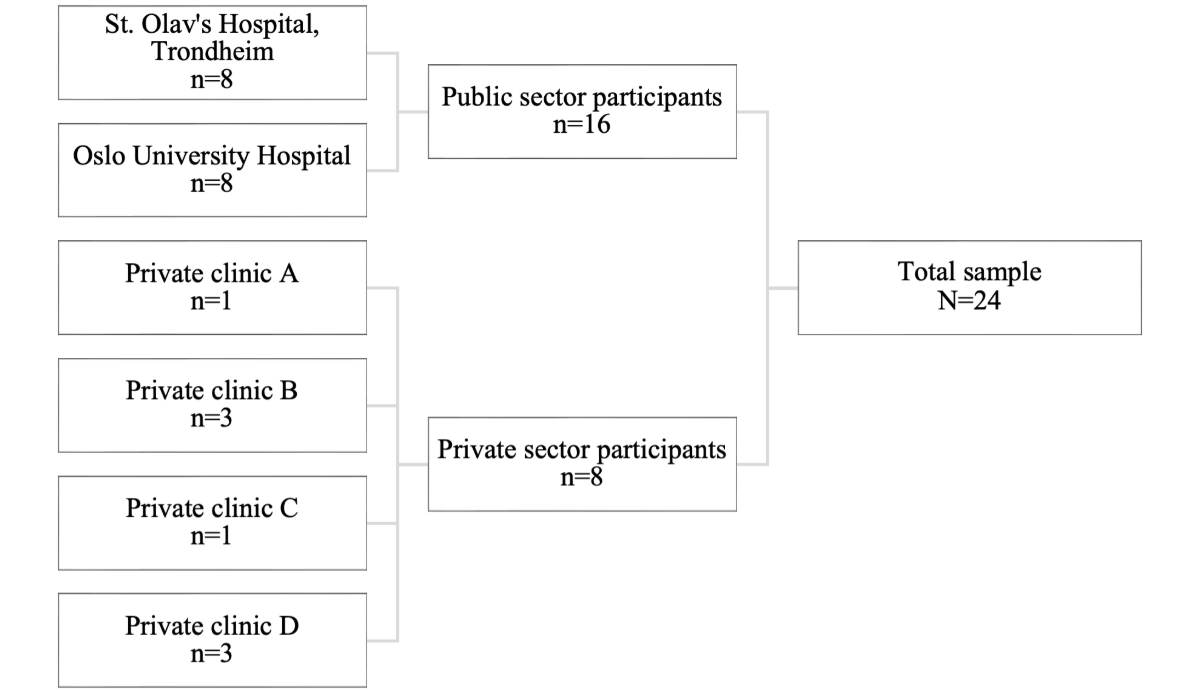
Chart of recruitment sites and study participants.

**Figure 2:**
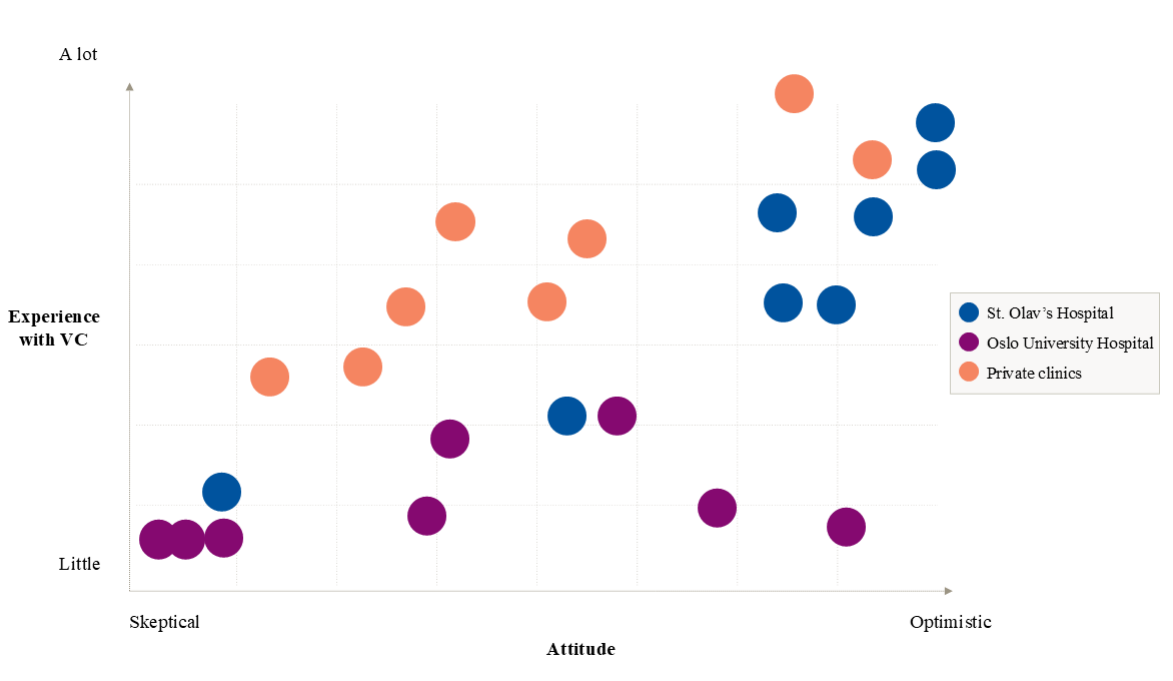
Early qualitative mapping of the experiences and attitudes of participants. VC: video consultations.

A total of 24 mental health professionals participated in our study (Figure 1 and Table 1), representing different demographic characteristics, geographical catchment areas, professional backgrounds, types of services, and target patient populations, as well as affiliation to either the public or private health sector. From the earliest phases of the analysis, we observed great variation in the participants’ attitudes toward VC and in the amount of personal experience with VC as a therapy format. Figure 2 is a result of early familiarization with the data material (Multimedia Appendix 3). The figure provides a simplified overview of the 3 subgroups in regard to levels of VC experience and attitudes. The figure is based on subjective and exploratory screening of the main tendencies in the participant accounts. The most extreme values on each of the axes represent the most extreme participant views identified in this study sample, and the position of each data point is relative to the others. Taken together, the distribution of data points in the figure suggests that there is a tendency that VC experiences and VC attitudes are related and that the relationship between the two concepts is stronger at more extreme levels. Moreover, the figure indicates differences in the amount of experience with VC between the 3 subgroups. Nevertheless, a continuum of attitudes is represented in each group. Early screening of attitudes in relation to participants’ characteristics indicated that variations in attitudes did not systematically follow affiliation with the public or private sector or the type of service the mental health professionals worked with.

**Table 1:** Participant characteristics (N=24).

Characteristics		Values
Participant age (years), mean (SD; range)		45.5 (9.9; 31-65)
**Sex assigned at birth, n (%)**		
	Female	17 (71)
	Male	7 (29)
**Role, n (%)**		
	Clinician	16 (67)
	Leader (few clinical tasks in everyday workflow)	3 (12)
	Combined (significant amount of clinical tasks in everyday workflow)	5 (21)
**Professional background, n (%)**		
	Specialist in clinical psychology	9 (38)
	Licensed clinical psychologist	5 (21)
	Psychiatrist	4 (17)
	Resident doctors in psychiatry	3 (12)
	Psychiatric nurse	2 (8)
	Nurses	1 (4)
**Type of mental health service, n (%)**		
	Open booking/no referral	8 (33)
	Psychotic disorders	5 (21)
	General psychiatric services	4 (17)
	Short-term general psychiatric services	3 (12)
	Bipolar disorder	3 (12)
	Currently not working in a clinical service	1 (4)

Through the following phases of the reflexive thematic analysis, we constructed a set of 6 themes, with each theme belonging to a distinct level of abstraction (Textbox 1).

**Themes from the reflexive thematic analysis listed according to level.**

**Meta level**

- Theme 1: meta-perspectives on the digitalization of therapeutic rooms

**Service level**

- Theme 2: the “how” of service integration

**Therapist culture level**

- Theme 3: challenging therapist culture

**Clinical level**

- Theme 4: negotiating the limits of the digital therapy room
- Theme 5: creating clinical value from the video format

**Practice level**

- Theme 6: adapting techniques and technology in digital therapy

### Theme 1: Meta-Perspectives on the Digitalization of Therapeutic Rooms

The first theme connects the practice of VC to the overarching digitalization trends in society and health care services. The participants demonstrated opposing underlying values that naturally influenced their standpoints at lower levels (ie, the 5 other themes). Interestingly, these meta-perspectives did not seem to follow other key characteristics of the participants, such as age, gender, private or public workplace, clinic, or severity of the condition of their patient population.

Some viewed the increased attention paid to VC in mental health care as a symptom of the fast-paced digitalization of our communities, which reduces arenas for healthy ways of experiencing and relating to others. Using this rationale, VC practices contribute to an unwanted development toward more individualism and isolation. Thus, there is a need to safeguard some of these analogue spaces, especially traditional therapy rooms, which might be particularly important.

Personally, and on behalf of others, I find it very exhausting to interact so much digitally. It has an overhead cost, whose extent I think we don’t fully understand yet.

B4, resident doctor in psychiatry, public hospital

Digital (encounters) enhance the extremely rational, intellectualizing way we relate to ourselves and others, which I believe results in more psychopathology.

D1, specialized psychologist, private clinic

The opposite viewpoint places digitalization in relation to new dynamics in the distribution of power and agency between patients and services. Since offering digital treatment can increase access to care and help balance the relational asymmetry of health care services, this viewpoint treats the expansion of therapy alternatives as a moral obligation.

They experience a higher degree of autonomy and control when they meet me at a screen’s distance.

A1, psychiatrist, public hospital

We deprive some patients of the opportunity to show up—patients who would otherwise struggle because they must enter our arena. There is already an imbalance—an asymmetrical relationship. Therefore, I think we owe it to them.

A2, specialized psychologist, public hospital

### Theme 2: The “How” of Service Integration

The second theme captures the diversity in VC integration strategies and emphasizes the relevance of exploring creative possibilities of VC use further. The data material contained descriptions of various service models involving VC in different ways, serving different purposes. See Textbox 2 for an overview of the service models identified in the study. The list demonstrates that using VC does not mean one thing. Rather, VC can be adapted to the specific needs of different clinical contexts. In practice, the participants often applied a combination of elements from the listed service models. We identified the most evident differences between the sectors in these dimensions. Some models were more available or exclusively implemented in the private sector, such as the digital hub of specialists. The hub allows personalization on several levels.

**Service models that integrate videoconferencing identified in the data material.**

**Only digital**

- All consultations in a course of treatment are digital. One private therapist worked exclusively with video consultations (VC) from home. One public clinician worked with a fully digital intervention for relatives.

**Digital first session**

- All new patients attend their first session through VC. Later, they decide on the format. Used in a public service for bipolar disorder that served many patients referred directly from emergency wards.

**Hybrid strategy (relational booster rationale)**

- Hybrid courses of treatment where traditional consultations comprise a minority of meetings and are used to strengthen the therapeutic relationship. Patients typically live far away.

**Hybrid strategy (content rationale)**

- Hybrid courses of treatment where the format depends on the session’s content. For instance, parts of psychiatric assessments where only verbal reports are needed can indicate a VC session.

**Hybrid strategy (practical rationale)**

- Hybrid courses where there is a preplanned strategy of using both formats to reduce travel or absence from daily activities. Target patients may have a network and activities they attend regularly.

**Hybrid strategy (symptom-based rationale)**

- Hybrid courses where anxiety-related symptoms are barriers to receiving services, and therapy starts off as VC, while the proportion of physical meetings gradually increases based on symptom reduction.

**Hybrid strategy (staff flexibility rationale)**

- Clinicians work from home on days when only digital appointments are scheduled to increase variety, flexibility, and satisfaction with work.

**Ad hoc hybrid courses (short-notice changes)**

- Traditional sessions may change into digital ones at short notice due to patients experiencing unanticipated events or problems with executive functions. VC sessions secure continuity.

**Open booking (only private clinics)**

- A client-centered service that allows anyone to book a VC session at a suitable time with the therapist they prefer, typically directly through a website. Some clinics also offer different durations of sessions.

**Digital hub (only private clinics)**

- Clinics provide information about therapists’ competencies and preferred therapy orientations, offering a menu of therapeutic alternatives from which clients can choose the best match for their needs.

Here, you can choose someone who matches where you are in the therapy process, what your goals are, and how you like to work. I think this leads to faster improvement compared to going to someone at random.

D1, specialized psychologist, private clinic

Furthermore, working independently with more flexible opening hours in a private clinic allows more staff flexibility for practitioners:

It is nice to have some days at home, deciding for myself how I want to structure the day. I might have a consultation at nine, the next consultation at one. Then one at six, and the last one at eight. Depending on what the client needs. And meanwhile, I can just live my life.

E2, psychologist, private clinic

Hybrid, preplanned strategies for delivering therapy for practical and symptom-related reasons were applied across the sample. However, a few therapists also talked about using traditional sessions to boost the relational quality of mainly digital courses of treatment, where the effect of the physical conversation would be maintained for a while:

... then we can keep leveraging from that (physical) booster session in the other (digital) conversations.

E3, specialized psychologist, private clinic

An insight from this dimension was that the clinicians’ presentation of how a VC service could work out is important, as it can shape patients’ expectations. One of the participants worked with a completely digital support service for the relatives of patients with severe mental illness. The clinician described how the relatives accepted the digital service they were offered and that there were no requests for in-person meetings:

The thing about the (digital service to relatives) conversations is that they are video-only. Right? Even from the first session. So, you go into it with the expectation that we will only meet on video.

B5, psychiatric nurse, public hospital

### Theme 3: Challenging Therapist Culture

The third theme is based on the participants’ descriptions of VC as a deviation from tradition and something that challenges assumptions established in professional culture. Some participants described video practice as a threat to therapists’ integrity, associating it with something less therapeutic than traditional therapy. Others said that VC could trigger a feeling of helplessness and less confidence in their role as therapists, a threat to their professional identity.

It’s kind of taboo in a way ... I think some view it as somewhat poorer. That you don’t really connect with the patient properly, when you don’t meet and look this person properly in the eyes.

B7, nurse, public hospital

There is sometimes a need to initiate acute interventions, and then you don’t necessarily get in the right position to act ... you feel more helpless on video.

B2, resident doctor in psychiatry, public hospital

Nevertheless, several participants challenged their preconceptions through experience and developed new, accepting approaches to VC. They reflected on the norms and myths of psychotherapy and identified culture-based assumptions about digital therapies:

If people were used to sitting at home and talking, and that was the standard, I am pretty sure going to an office would have felt strange.

A6, specialized psychologist, public hospital

It has become imperative that the patient comes to the outpatient clinic and stays there for 45 minutes. I do not have any evidence to state that it is very effective or useful for either the patient or us. ... It is deeply ingrained in healthcare culture. Things like video consultations are not part of education programs. ... This influences what one thinks as a clinician.

A7, specialized psychologist, public hospital

As observed early in the familiarization process of the dataset, there seemed to be a trend that the most experienced VC therapists held more positive views, and vice versa. However, we also observed that private practitioners with more experience than employees from public hospitals conveyed diverse opinions. Interestingly, in private clinics, patients decide on the therapy format themselves when they book a consultation. Thus, some private practitioners may practice an even amount of VC therapy, even if they subscribe to a therapist culture that favors the physical therapy space.

### Theme 4: Negotiating the Limits of the Digital Therapy Room

Theme 4 demonstrates the contrasts in professional opinions and rationales regarding the clinical purposes for which VC is suitable. All participants acknowledged that the digital therapy room was less appropriate when patients lacked social contact and activities in their daily lives or needed exposure to work with avoidance. Apart from this, their reflections on the feasibility of different therapy contents varied.

**Figure 3:**
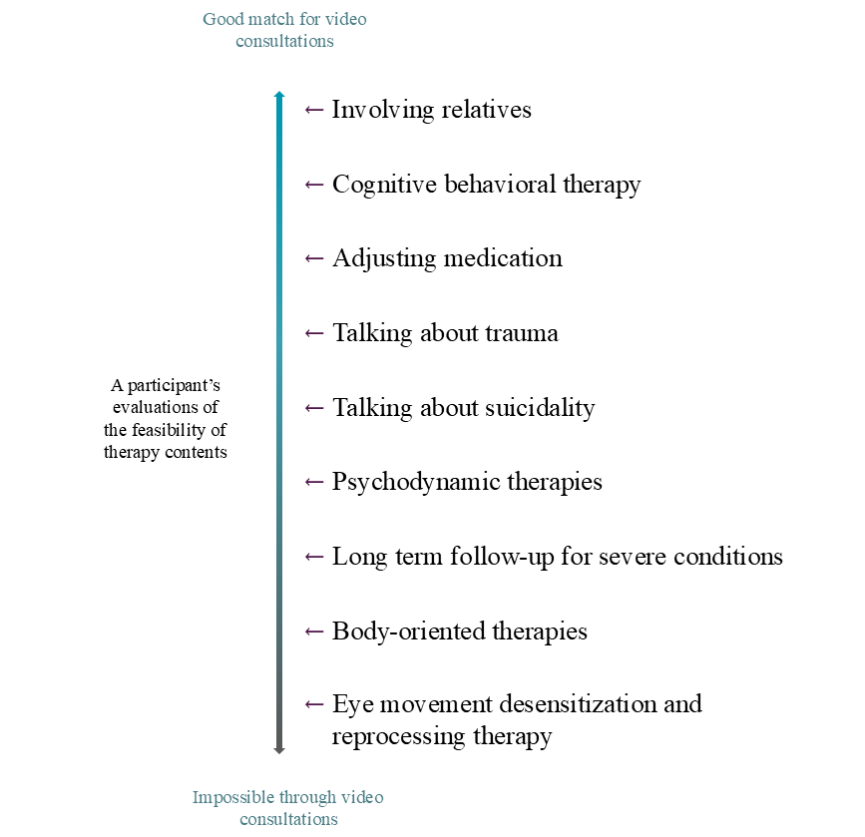
Illustration of a typical continuum of feasibility concerning different therapy contents when delivered through videoconferencing, based on 1 participant.

Figure 3 illustrates the continuum of the perceived feasibility of different therapy contents when delivered through VC, based on a participant who worked within the cognitive therapy orientation. The case presented in the figure demonstrates how participants typically had clear logic and presented a continuum of feasibility, defining the limits of VC. From the viewpoint in this case, eye movement desensitization and reprocessing therapy (EMDR) is the least feasible intervention using VC.

However, others expressed contrasting opinions. These perspectives on feasibility are demonstrated in the following extracts:

There are certain things that are not easy to do on video—that cannot be done on video. For example, trauma therapy with EMDR. It is completely impossible.

A3, psychiatrist, public hospital

I might think that EMDR works better digitally than other forms of therapy, because there are very few words.

D1, specialized psychologist working with EMDR in traditional sessions, private clinic

Similarly, while most participants talked about patients with psychotic disorders as the patient population least probable of experiencing value from VC, one of the clinicians working with this patient group remarked,

I think the patient can often surprise the clinician in that area, and they can often be better (than what the clinicians expect).

B7, nurse, public hospital

Finally, some mental health professionals demonstrated the importance of understanding the difference between the feasibility of carrying out interventions and the actual therapeutic value that is realized. In the context of services aimed at patients struggling with severe diagnoses and complex life challenges, a VC may not achieve the same outcomes as in-person contact. A participant stated that services and clinicians are at risk of misleading themselves in the following way:

“Now that we have digital tools, we actually get in touch with the patient. The patient attends the appointments. We are now in a position where we can talk to this person.” However, the patient just sits there in their municipal apartment, isolated, without needing to go out. “But at least we get in touch with him!” I imagine that we might end up just believing that we have done something good.

B4, resident doctor in psychiatry, public hospital

### Theme 5: Creating Clinical Value From the Video Format

The fifth theme revolves around the process of realizing therapeutic value from VC. The data material contained examples demonstrating that the video format, in some cases, comes with unique additional clinical benefits:

I have many clients whom I have never met in person. However, it was particularly interesting that with those I had met in person, I felt that more happened when we could talk on video, and I experienced more therapeutic breakthroughs with them after we started with video consultations. I found that a bit interesting.

E2, psychologist, private clinic

Several clinicians explained that the practical benefits of the video format mediated some of the extra therapeutic value. When patients do not need to spend much time travelling or spend energy overcoming practical obstacles and stress related to a physical meeting, more resources may be available for the purpose of the therapy session. Moreover, remote sessions often come with proximity to family members, which can result in more effective therapeutic processes:

They might have to sit on a bus for two hours each way, and then the entire consultation would end up talking about how terribly frustrating it had been. When you have a video consultation, you can really concentrate on what the underlying problem is.

A3, psychiatrist, public hospital

I had the opportunity to do things more quickly. I found out that it might be a good idea to talk to (the patient’s) relative just to calm the situation and asked if that relative was present at home. The patient said yes, and then I got 15 minutes with the relative right away. And then that situation was solved. It wasn’t an issue anymore.

A6, specialized psychologist, public hospital

Another application of VC is as an aid to regulate patients’ level of activation. Facilitating a safe, less confronting therapy environment reduces the level of activation, providing patients with more opportunities to take part in and experience the effects of emotionally challenging therapeutic interventions:

If there is a really difficult conversation with a client where we are going to talk about our alliance, I might take the client out of the therapy room to help downregulate feelings, not having to maintain eye contact. We can use video to do the same. Sometimes, when we want feelings to be more intense, I prefer to be in the office.

E1, psychologist, private clinic

### Theme 6: Adapting Techniques and Technology in Digital Therapy Sessions

The sixth theme is placed at the concrete level of therapeutic practice, covering pragmatic adaptations of therapy interventions and technical aspects of the technologies used for VC. One clinician always wore headphones during VC to optimize the dynamics of the conversation, rather than using the speakers:

I get the sound directly in the ears instead of it coming from speakers. That makes a difference.

E3, specialized psychologist, private clinic

Another clinician talked about how basic VC therapy skills provide great opportunities to carry out therapeutic interventions that traditionally depend on the physical environment of the therapy room. This clinician emphasized the importance of careful verbal guidance and attentive assessment of the patient’s needs during VC:

I don’t feel (that the video format) is an obstacle because I can guide them and see if there’s something they need, find a slightly better chair to sit on, or if they have something they can hold in their hand, or if they’re working on regulating themselves a bit. Grab a pillow or something like that. They can do that at home as well.

D3, specialized psychologist using body-oriented methods, private clinic

Several participants shared the opinion that there is potential for improvement for future VC practices. Some said that simply improving the information about VC directed at patients could strengthen implementation. Others talked about potential improvements in the technology; for example, how AI might enhance the liveliness of VC. Finally, multiple participants talked about the lack of safe and clinically validated software that can mimic whiteboards and paper-based tools that are typically used in traditional assessment and therapy sessions. Such technology already exists and is integrated into VC systems used in other professional contexts, but is not available for clinical use in mental health care.

If we get an AI as a video chat function, then we could have more visual dynamics. And I think these dynamics will elevate the experience.

B2, resident doctor in psychiatry, public hospital

If there were a function where you could incorporate questionnaires or some kind of interactive board in the video call (...) That could perhaps also address some challenges related to forms and documentation.

A8, specialized psychologist, public hospital

## Discussion

### Principal Results

This study explores experiences, attitudes, and current debates related to the role of VC in mental health care and challenges preconceptions identified in our own research environment and beyond. To our knowledge, this is the first qualitative study to explore the experiences and attitudes of mental health professionals across public and private settings on the topic of VC. Moreover, the study included perspectives from a broad selection of services, ranging from those targeting bipolar and psychotic disorders to on-demand services targeting clients with milder conditions and not requiring referrals. Our study identifies differences in VC implementation, experiences, and professional attitudes that support the existing literature [31,32,42,43]. Contrary to the authors’ expectations, key characteristics such as public and private sector affiliation and symptom severity of the target patient populations did not seem to shape the participants’ views to a significant degree. Building on this, our study points out important areas to direct future efforts to increase the adoption and impact of VC.

Primarily, our findings demonstrate that higher-level aspects, such as societal values and cultural perceptions, are important in shaping professional attitudes. Psychotherapy and the therapist role are defined through the cultural value systems in society and in professional environments [70]. Thus, fundamental changes in mental health care services, such as the digitalization of the delivery of therapy, can be experienced as a cultural deviation from traditional models of psychotherapy [71,72]. In accordance with this notion, several participants talked about how traditional ideas about psychotherapy weaken the credibility of VC, although many forms of VC-delivered psychotherapy are supported by robust evidence [5-9]. This tendency indicates a lack of cultural familiarity with digitalized treatment and a lack of knowledge about the evidence-based applications of VC.

While the status of VC in clinical environments remains unclear, polarized approaches have been developed. In our study, the participants with the most critical attitudes typically conceptualized VC as part of purely digital, fast-paced, cost-reduction–focused services and, thus, a symptom of an unwanted development in health care. Research on the resistance to digitalized mental health services has identified similar viewpoints of clinicians who experience that technology conflicts with fundamental humanistic values in mental health care, resulting in resistance and stigma related to the digitalization of therapy [72]. This approach facilitates an all-or-nothing view of digitalized services instead of a nuanced perception of VC as a flexible tool that can be integrated in various ways in hybrid services, a model that is becoming increasingly applied in the literature [25-27].

Interestingly, even among the participants who held the most positive attitudes, we found conflicting views on whether VC is feasible for patients with severe conditions. These clinicians described the patient group as vulnerable and conveyed that it was their professional responsibility to protect these patients accordingly. However, adjustments to the service alternatives based on the vulnerable status of patient groups may not always result in better outcomes for the patients [73]. Furthermore, the exclusion of such patient populations may be based on stigmatic perceptions of the patients, which have been found to be relatively frequent among clinicians working in mental health care services [74,75]. Differentiating the treatment offered based on patients’ diagnoses may preserve such preconceptions. Although the existing evidence pertaining to VC in services targeting these populations is still scarce and calls for more research [15-19], we encourage an inclusive and personalized approach and advocate refraining from excluding patients based on diagnosis alone.

Importantly, the education of mental health professionals plays a fundamental role in defining the scope of psychotherapy and conceptualizing VC. Currently, VC is mostly absent from the training of mental health personnel, which may promote more trust in traditional cultural perceptions of therapy and the limits of VC. Moreover, mental health professionals report low self-efficacy and a lack of training and support in clinics [14,35,36,47,48]. At the same time, the research field continues to produce new knowledge about VC, such as through studies focused on adapting basic therapeutic skills to the video format and studies on how we can maintain a feeling of connectedness in video sessions [10,14]. In addition, the body of literature covers adaptations of interventions within therapy orientations that are often closely associated with traditional settings and that emphasize sensory experiences and the atmosphere of the therapy room, such as psychodynamic transference [11], emotion-focused therapy exercises [12], and EMDR [13]. Our findings encourage the inclusion of knowledge about VC in the basic training of professionals to ensure that VC is incorporated from an early point.

We also found that the amount of personal experience with VC plays an important role in shaping attitudes, as it directly challenges established cultural assumptions. Several participants expressing optimistic attitudes stated that they had not been able to perceive VC as feasible and valuable before they had practiced it themselves and adjusted their preconceptions. These observations partly align with previous research showing that more experience with VC is associated with positive attitudes and less self-doubt as a therapist [49-51]. These findings are supported by research suggesting that one of the main drivers of adoption is clinicians’ own personal experiences, proving that digital tools can result in clinical benefits [76]. Together with the literature, our findings emphasize the importance of personal experience to perceiving the value of VC. Thus, we suggest that services become aware of this tendency, demonstrate the beneficial effects for both service users and providers, and develop incentives for clinicians to integrate VC in their practice.

Finally, our study presents a range of creative and innovative therapy practices and service designs, suggesting that there is potential synergistic value in sharing local insights between professional environments. Several of the service models identified have been implemented only to a very limited degree across the clinical sites of the study, such as the digital hub or digital first sessions (see Theme 2 in the Results section). Some of these service designs have been mentioned in the literature but have not been tested in public settings in Norway [27]. Using insights and service models from both the private sector and innovative public sites may be valuable in future decision-making regarding service designs in public health care. Interestingly, few innovative activities revolve around the technologies used for VC, and clinicians report that the platforms they use lack the basic tools used in traditional therapy [24,41,46]. This indicates that there is room for improvement and further innovation in terms of clinical VC software, which has barely changed since the introduction of VC in mental health care.

### Limitations

Although the method chosen for this study does not aim to produce generalizable findings, and although our findings can still be transferable, we wish to explicitly acknowledge that the composition of our sample may have moved the analytical focus and the results in a more optimistic and progressive direction. For instance, several participants recruited from one of the public hospitals were involved in research and development, while two were involved in digitalization projects. We are also aware that the participants who decided to participate might have had more positive attitudes toward the topic of the research project than those who did not want to participate. This, combined with the authors’ optimistic attitudes and roles in other digitalization projects closely related to the topic of this study, possibly influenced the angle we chose for this paper, which encourages digitalization in mental health care. Another limitation relates to the practical transferability of findings produced in this study in an international context, as all participants were recruited from Norwegian health care sites. Furthermore, the analysis is not exhaustive in discussing aspects that have an impact on the participants’ viewpoints. For instance, regarding the private actors’ contextual characteristics, the paper discusses the impact of incentives for practicing VC and whether VC use is voluntary or not to a very limited degree. An important research-related issue resulting from such differences in public and private sector contexts revolved around the recruitment for this study. Participants from private clinics received economic compensation of about US $110 because of slow recruitment due to the nature of the private practice financial model. Lost activity with paying clients results in reduced income, which is a barrier related to participation in research projects. The other participants did not receive similar economic compensation since they were all employed at university hospitals, where participation in research and development projects is integral. Future research should explore the effects of different organizational conditions in detail, which may be of great importance in developing attractive workplaces for mental health professionals and sustainable hybrid services.

### Conclusions

This qualitative exploration emphasizes the multidimensional nature of professional perspectives on VC. Our findings underline how overarching societal and cultural ideas influence professional attitudes, logic, and, thus, clinical practice. Our ambition is that the paper challenges preconceptions about digitalized therapy and that it points to useful strategies for targeting problems concerning the limited adoption of VC. Educational institutions and services must promote a nuanced, evidence-based concept of VC and demonstrate benefits for both service users and providers. In addition, incentives for mental health professionals to practice VC should be developed and presented. There is unrealized potential in applying existing therapy adaptations and hybrid service models established in separate parts of health care systems, particularly for public clinics. In addition, there is room for innovation with regard to service designs that integrate VC and the technology platforms used to create unique value in hybrid services and to increase the attractiveness of the VC format in mental health care.

## Data Availability

To protect the anonymity of the participants, the qualitative dataset generated during this study will not be available to readers. More information concerning the study settings is available from the corresponding author on reasonable request.

## Appendix

Multimedia Appendix 1 Supplementary figures.

Multimedia Appendix 2 Interview guide.

Multimedia Appendix 3 Detailed description of the analysis.

## Abbreviations

EMDR: eye movement desensitization and reprocessing therapy
TSD: Services for Sensitive Data
VC: video consultations
