# Supplementary material for: Exploring Video Consultations Across the Public and Private Sectors in Norway: Semistructured Interview Study
Source: JMIR Hum Factors. 2026 Jan 26;13:e80812. doi: 10.2196/80812 (PMC12887556; doi:10.2196/80812)
Supplement: Multimedia Appendix 1 [file humanfactors_v13i1e80812_app1.docx]

**Figure S1.** Communication flow for recruitment at St. Olav’s Hospital.

**Figure S2.** Communication flow for recruitment at Oslo University Hospital.

**Figure S3.** Communication flow for recruitment at private clinics.
