# Supplementary material for: Exploring Video Consultations Across the Public and Private Sectors in Norway: Semistructured Interview Study
Source: JMIR Hum Factors. 2026 Jan 26;13:e80812. doi: 10.2196/80812 (PMC12887556; doi:10.2196/80812)
Supplement: Multimedia Appendix 2 [file humanfactors_v13i1e80812_app2.docx]

**Interview guide (translated from Norwegian)**

- What is your role at your workplace?
  - Clinician working directly with patients?
  - Leader responsibilities?
  - Combination?
  - Professional background and education
  - Main tasks
- How do you and the clinic you work at approach video consultations?
  - Who are offered video consultations?
  - Who ask for video consultations?
  - What types of consultations, therapy orientations, and patient populations, are suitable for video consultations? Which problems can be solved using video consultations? Which topics can be brought up?
  - What is unsuitable for video consultations?
- Your workplace
  - Is the use of video consultations a individual choice?
  - How do you and your collueages talk about video consultations versus in-person encounters?
  - What characterizes a collueague who chooses to use video consultations?
  - How does the management talk about video consultations and digital interventions?
  - Does your workplace have specific routines related to the use of video consultations?
  - Has your workplace arranged video consultation training?
- How do patients participate in video consultations?
  - Smartphone? Computer? A blend? Only audio?
  - Outside, at work, in their car, from home?
- How do you think patients experience taking part in video consultations?
  - Positive, negative?
  - Have you received any feedback?
  - How do you discuss the video consultation versus in-person alternatives of treatment delivery with patients?
- How do you experience working with patients through video consultations?
  - What is different from in-person encounters?
  - Do you have any negative experiences?
  - What does video consultations add to the course of treatment?
  - Do you use tools during video consultations?
  - Do you miss anything?
  - Do you experience barriers to using video consultations?
