# Supplementary material for: Exploring Video Consultations Across the Public and Private Sectors in Norway: Semistructured Interview Study
Source: JMIR Hum Factors. 2026 Jan 26;13:e80812. doi: 10.2196/80812 (PMC12887556; doi:10.2196/80812)
Supplement: Multimedia Appendix 3 [file humanfactors_v13i1e80812_app3.docx]

**Detailed description of the reflexive thematic analytic procedure**

***Phase one.*** In the first step in the data familiarization process, MS went through all the AI-transcribed texts and corrected them in accordance with the original audio recordings. Complete anonymized transcripts were uploaded to NVivo. MS reread each transcript and created electronic memos for each transcript, summarizing core statements that seemed directly relevant to the study aims. To facilitate further reflection on the dataset, MS created a graphical illustration organizing the participants in relation to each other based on the memos (see Figure 2 in Results).

***Phase two.*** Using NVivo, MS coded the transcripts using mainly semantic codes to adhere closely to participants’ formulations. With this coding strategy, we aimed to capture concrete practices, experience-based insights, and general reflections concerning VC from the viewpoint of the participants. Taking a more active coder role, MS also created some additional codes at a higher level. This strategy ensured that important dimensions that were not described explicitly by the participants were included in the later analytic phases. MS moved to phase three when she considered all text elements relevant to the research aims to be coded.

***Phase three.*** To initiate the process of generating candidate themes, MS collapsed groups of codes and added overarching titles to the clusters in NVivo. This resulted in 19 topic-like themes, such as ‘travel distances’ and ‘well-being of clinicians’. MS combined some of these early candidate themes and removed others, reducing the total number of themes and adding complexity. MS drafted a thematic map consisting of two levels, overarching themes and subthemes. This structure captured most of the elements that were included in the final report but looked very different from the final set of themes and its organization.

***Phase four.*** MS started the process of reviewing and revising the candidate themes and the thematic map. MS studied both the specific data extracts related to each theme and scanned the entire data set to evaluate and further develop the analytic output. In this phase, MS consulted SRA for sense checking, ensuring reflexivity and sparking creative thinking. The discussion resulted in a new structure of abstraction levels that organized the themes in a more meaningful way. The levels comprised meta, service, clinical, and therapeutic practice levels.

MS then generated a new level to include aspects related to professional culture and split the clinical dimension into two, resulting in one theme about feasibility and one about clinical value. The decision to assign a heavier weight to the clinical level was influenced by feedback from user representatives with lived experience, which was conveyed in the planning phase of the study. The representatives advised the project group to include open-ended questions about how the mental health professionals perceived patients’ experiences in the interview guide and to put an analytical emphasis on clinical value. The result of the fourth phase was six themes.

***Phase five.*** MS adjusted the set of themes several times, with the aim of producing meaningful and logically structured concepts with interesting names. During this process, MS had discussions with SRA, HMI, KJK, LNS, and KLR to obtain feedback regarding the new multilevel theme structure of themes. Decisions regarding both the title, scope and position of each theme were a pronounced challenge during this phase. This especially applied to the position of the themes concerning therapist culture, clinical value, and therapy rooms. The processes of phase five were highly interconnected with the writing process of phase six.

***Phase six.*** MS briefly initiated report writing at the very beginning of the data collection to establish a base for a dynamic process. MS produced slightly more text at the beginning of phase three and started writing more intensively in phase five. The writing process continuously challenged how the themes were defined and described. When the themes were considered finalized, the structure of themes set the direction for the report. Decisions concerning which main elements of meaning to include and which quotes communicated these concepts most efficiently in the report continued to shape the themes. Thus, the results section of this paper has undergone significant modifications in parallel to ongoing reflexive thematic analysis.

***Researchers and reflexivity***

The authors are colleagues at Oslo University Hospital. All authors contributed to designing the study, recruiting institutions and participants, and writing. The first author, a female PhD candidate trained as a licensed psychologist, led the analysis and write-up of this paper under the guidance of the other authors. Several authors had a clear expectation that private actors would have more optimistic views compared to the participants employed in the public sector, which influenced how the researchers designed the study and approached the data material. Moreover, all authors are involved in research and innovation projects focused on the digitalization of healthcare services and, in general, hold relatively optimistic views regarding both the use of technology in treatment and the innovation of services. Thus, the authors may be impacted by long-term goals related to documenting positive outcomes from the digitalization of services. We recognize that these aspects are present throughout all the components of this study.
